# The Evolution of the Modern Hospital

**Published:** 1915-03-06

**Authors:** B. Burford Rawlings


### THE EVOLUTION OF THE MODERN HOSPITAL.*

### II.—The Nightingale School and a New Epoch.

By B. BURFORD RAWLINGS.

Years before, Florence Nightingale had drawn attention to the grave deficiencies of hospital nursing, and her devotion at the time of the Crimean war had impressed the public mind so deeply that a large sum of money was subscribed and presented to her in token of the nation's gratitude. It was a characteristic act, and a proof of her transparent sincerity, that she immediately applied the gift to the establishment of a School of Nurses. This school was associated with St. Thomas's Hospital, and we are told that it was to be "governed by principles formulated by Miss Nightingale."

Events have moved rapidly since those days, and no one would have admitted more readily than Miss Nightingale herself that the principles she proceeded to formulate would be a long way from finding acceptance now. So far as they went, they were admirable, but they fell short of much that present opinion regards as indispensable. The training of one year's duration she demanded has become a three years' course; and even after that long period of probation and the attainment of a certificate, the ardent nurse will often take up particular work in special hospitals in order that she may get abreast of the knowledge applied in the nursing of maladies not ordinarily admitted to a general hospital, or treated only until the acuter symptoms have abated.

Yet what Miss Nightingale demanded and in time obtained marked the awakening from sleep, and a daylight revelation of duty to the sick never previously so much as dreamed of. The good effects of the Nightingale School travelled far beyond the primary object, the training of nurses. The influences emanating from it stirred the dry bones of our hospital system and endowed medical charity with a spirit of humanity it had never before possessed. Thus was it eminently appropriate, as it would appear almost predestined, that the establishment of the School of Nurses should help to bring about vast changes in the domestic conditions of the hospital with which the school was incorporated, and, ultimately, of all hospitals. Like many other events big with consequences, it did not carry its full significance upon the surface, but looked at after a lapse of years, when all the lines of action starting from it can be traced to their source, who can doubt that the new departure initiated an epoch? It had planted a soul within the ribs of a defective and almost decadent philanthropy, and brought a warmth, a cleanliness, and a beneficence into hospital life whose wholesome harvest the sick are gathering in to-day.

Other hospitals were soon awakened, and began to place their houses in order. The training of nurses became a recognised duty at St. Bartholomew's, the London, Guy's, the Middlesex, and indeed at all the great general hospitals of London and some of the special hospitals, together with many of the provincial hospitals and infirmaries, and it is a remarkable fact that wherever the new ideas penetrated in respect of nurses and their training, there other improvements followed, and a new and happier era in the domestic history of the institution opened.

To those gifted with insight this was no more to be wondered at than gainsaid. In the ancient régime of hospitals the potent influence of the gentlewoman was unknown. The matron, usually a respectable personage of the social status of an old-world housekeeper, whose afternoon dignity was well portrayed in a wondrous dress of black satin, measured her sense of duty by no very exalted standard. Propriety meant absence of scandal; the externals were the essentials, and, as what cannot be helped must be endured, she was considerately blind to, even if she appreciated, the shortcomings of the ignorant women who worked under her, well satisfied if her "nurses" stopped short of drunkenness when on duty and were not so openly neglectful and unkind in their relations with the patients as to invite observation.

Looked at exclusively from a patient's point of view, a hospital possibly can never be made an ideal habitation for a sick person. The want of privacy, the sense of being but a sojourner, the unavoidable traffic, the proximity of other sufferers, and the distressing scenes enacted by night and day, not to write of other sources of discomfort, are drawbacks from which no practical provision can wholly preserve. But to what extent their consequences may be mitigated, a visit to a present-day ward will show. The apartment is as bright and inviting as willing hands can make it. Comfort and cleanliness are in evidence; the atmosphere, both material and moral, is sweet, and even the elegances of life find a place. What a contrast to the dim, close, roughly equipped chamber of half or even a quarter of a century ago! The ward is now lighted electrically, and every bed is provided with a lamp of its own for use when medical examinations are made. The tables, which at one period held little besides bottles of stock mixtures, other portentous and more offensive emblems of the sick room, and litter, are now graced with flowers. Not the simple, florid, solid, and half-withered posies which friends of country patients brought from cottage gardens, and somewhat impatient attendants had pushed, still tightly tied, into jam-jars or other supernumerary articles of earthenware. These were welcome enough to the patients to whom they were sent as messengers from home, and all the sweeter in their sentiment if the worse in their materiality for their long journey in perspiring hands. Now we find rare and exquisite blooms, arranged with good taste amid a profusion of palms and ferns, perhaps the pick of the neighbouring florist's, and not infrequently bought at the cost of kindly and cultivated nurses.

* Previous article appeared on February 20.

